# Acute Cardiovascular Effects of Turkish Coffee Assessed by VO_2_ Test: A Randomized Crossover Trial

**DOI:** 10.3390/nu17050823

**Published:** 2025-02-27

**Authors:** Nour A. Elsahoryi, Mohammed O. Ibrahim, Omar A. Alhaj, Fadwa Hammouh

**Affiliations:** 1 Department of Nutrition, Faculty of Pharmacy and Medical Sciences, University of Petra, Amman 1196, Jordan; omar.alhaj@uop.edu.jo; 2 Department of Nutrition and Food Technology, Faculty of Agriculture, Mutah University, Karak 61710, Jordan; mohammedomar@mutah.edu.jo; 3 Department of Nutrition and Health Psychology, Faculty of Health Sciences, American University of Madaba, MR62+F79, Madaba 11821, Jordan; f.hammouh@aum.edu.jo

**Keywords:** Turkish coffee (TC), bioactive compounds, acute physiological effects, cardiovascular response, metabolic parameters

## Abstract

Background: Turkish coffee (TC), a traditional unfiltered coffee preparation method, contains unique bioactive compounds due to its specific brewing process. While TC’s cultural significance is well-documented, its acute physiological and psychological effects remain understudied. Objectives: This randomized, controlled crossover trial investigated the acute effects of a standardized TC dose (3 mg caffeine/kg body weight) on metabolic and psychological parameters in healthy female university students (n = 52, age: 20.25 ± 1.20 years). Methods: TC was prepared with a 1:1 ratio of medium and dark roasted Arabica beans. The chemical analysis showed the caffeine content to be 2.8 ± 0.3 mg/mL and the chlorogenic acid content to be 1.9 ± 0.2 mg/ml. Participants were randomized to receive either TC or water control, with a washout period of 8 weeks between treatments. Cardiovascular parameters, metabolic markers, and validated visual analog scales (VAS) were assessed at baseline, 60-, 90-, and 120-min post-consumption. Results: Heart rate showed significant time-dependent reductions in both groups (control: *p* < 0.05; TC: *p*-value < 0.01 at 60, 90, and 120 min vs. baseline). Heart rate dropped significantly in the Turkish coffee group, from 78.0 ± 10.2 bpm at baseline to 71.5 ± 9.5 bpm after 90 min (*p*-value = 0.002). Sleep scores also declined, from 4.38 ± 2.91 at baseline to 1.88 ± 1.45 after 120 min (*p* < 0.05), indicating a stimulating effect of caffeine. TC consumption significantly affected appetite sensations (*p* < 0.05) and sleep scores (F = 3.174, *p*-value = 0.029), with the TC group showing progressive reductions in sleep scores from baseline (4.38 ± 2.91) to 60 min (2.58 ± 2.04), and further decreases at 90 and 120 min. Conclusions: These findings suggest that TC exerts significant acute effects on cardiovascular function and psychophysiological parameters in healthy young females, potentially due to its unique phytochemical profile and preparation method.

## 1. Introduction

Coffee, in general, and Turkish coffee (TC), in particular, represents a large and complex group of biologically active compounds [1,2]. The extraction and preparation method are an important factor in determining the composition of these components such as caffeine and antioxidants [1,2]. Turkish coffee is distinguished by its unique preparation method from other types of coffee, which involves finely ground beans boiled without filtering, which ultimately gives it a distinctive phytochemical composition that distinguishes it from other coffee preparations [3,4,5]. The unfiltered brewing process of TC results in higher concentrations of bioactive compounds, including caffeine, chlorogenic acids, diterpenes (cafestol and kahweol), and various polyphenols [6,7]. Turkish coffee holds onto more cafestol and kahweol than espresso or filtered coffee—two compounds that may affect heart and metabolic health. In contrast, filtering coffee removes most of these fat-soluble diterpenes, which could lower their influence on cholesterol levels and overall cardiovascular function [6,7].

These compounds have been shown to have considerable biological activities, including antioxidant properties and protection of cells from damage by free radicals [8]. The molecular composition of TC is especially striking due to its way of preparation. Unlike the filtered coffee preparations, the prolonged boiling of TC, without filtration, leads to improved extraction of lipid-soluble compounds [6,7]. This unique extraction process results in higher concentrations of cafestol and kahweol, bioactive diterpenes that are largely removed by the paper filters used in most conventional coffee preparation methods [6,7]. At the molecular level, caffeine, TC’s primary bioactive compound, acts as an adenosine receptor antagonist with the potential to affect a variety of physiological parameters [9]. Although moderate coffee consumption (2–3 cups per day) seems to be safe from a cardiovascular point of view [10], the acute effects of TC’s concentrated bioactive compounds are controversial [2,6]. Most of the earlier studies investigating coffee’s cardiovascular effects have averaged long-term consumption [11,12,13,14], with a limited investigation of acute responses [5]. The underlying molecular mechanisms for the cardiovascular effects of coffee are versatile and multidirectional. Caffeine antagonizes adenosine, resulting in increased noradrenaline production, which leads to vasoconstriction [15,16]. These molecular interactions may affect heart rate (HR) by stimulation of the central nervous system [17]. Bioactive compounds contained in coffee also have regulatory actions on glucose metabolism [18,19] and energy expenditure via stimulation of thermogenesis and altering the expression of lipogenic enzymes [20,21,22,23]. Despite TC’s widespread consumption, particularly in the Middle East, research on its acute physiological effects remains limited. Individual caffeine tolerance may affect how the body responds to Turkish coffee. Regular coffee drinkers build some resistance to caffeine’s effects on HR, blood pressure (BP), and metabolism, which might reduce its immediate impact. Future research should take caffeine habits into account by grouping participants based on their usual intake to see if regular consumption changes the short-term effects of Turkish coffee. In Jordan, where coffee consumption reached 37.0 kt in 2020 (8.82% annual increase), adults average 33.9 mg/day of caffeine, primarily from unfiltered coffee varieties [24]. Understanding TC’s acute effects is crucial, given its unique molecular composition and preparation method. 

This randomized crossover trial aimed to determine the acute effects of a standardized dose of TC on physiological and psychological parameters in healthy university female students. Specifically, this study focused on the examination of cardiovascular responses (BP, HR) to metabolic parameters (fasting blood glucose (FBS), substrate oxidation), psychological responses using validated visual analog scales, energy expenditure, and thermogenic effects by elucidating TC’s acute effects. This provides insight into the biological activity of its unique phytochemical profile, contributing to our understanding of the molecular basis for the health implications of TC.

## 2. Materials and Methods

### 2.1. Experimental Design

This study was conducted on healthy, non-smoking female university students using a randomized crossover design. Participants had no neuromuscular restrictions, cardiopulmonary issues, or medications that could influence the study’s outcomes. To ensure an accurate assessment of cardiovascular and metabolic responses, individuals taking β-blockers, calcium channel blockers, or anti-diabetic medications were excluded.

Since the study involved Jordanian university students, the findings may not fully apply to Turkish populations. Genetic and cultural factors play a role in how coffee is metabolized and its effects on the body. Future research should explore whether Turkish individuals, who regularly drink traditional Turkish Coffee (Türk Kahvesi), experience similar physiological responses. Although the long-term health effects of regular coffee consumption—such as its benefits for heart and metabolic health—are well-documented [11,12,13,14], little research has explored the immediate physiological and psychological responses to drinking Turkish coffee. This study aims to fill that gap by examining how a single serving of Turkish coffee affects cardiovascular, metabolic, and psychological markers in the short term.

This study evaluated the acute effect of a single-dose TC on metabolic parameters like resting metabolic rate, HR, systolic and diastolic BP (SBP and DBP) in mmHg, oxygen consumption VO_2_ max in mL·kg^−1^·min^−1^, and VAS scale (0–10) for appetite, energy, mood, concentration, motivation, and fatigue. The randomization schedule for treatments was generated by computer software (G*Power version 3.1.9.7) prior to the beginning of the study. The study consisted of two one-day intervention phases with an 8-week washout period in a crossover design. One group of participants started with the ingestion of a dose-controlled TC of 0.136 g/kg (without cardamom or any other additions) of body weight in the morning after the baseline measurements, while the second group did not consume coffee but water instead; the interventions were crossed over during the second phase. The main aim of the study was to assess and compare the effect of a single dose of TC on metabolic parameters across the various intervention groups over the period of intervention. All the measurements involved in this study were performed in the nutrition clinic at the University of Petra. The investigators monitored safety throughout the duration of the study. They were supposed to report on all nonserious or serious adverse events relating to incidence, type, severity, and relationship to product intake.

### 2.2. Participant Recruitment

Power analysis was performed using G*Power 3.1, with α = 0.05, power = 0.95, and an expected effect size (Cohen’s d) of 0.6 calculated from previous studies [10,25], indicating a sample size of at least 25 participants per group. Each subject went through a randomized, crossover trial and, therefore, experienced both interventions separately in two sessions. In all, 25 healthy female university students, aged 18–39 years, were recruited from the general population using posters, emails, and personal advertisements. Potential volunteers were provided with a participant information sheet detailing the study and had ample time to consider participation. A contact phone number and email address were also available for any questions. Participants who expressed further interest were invited for a screening visit to determine their eligibility. Inclusion criteria were VO_2_ peak > 35 mL·kg^−1^·min^−1^, normal BP, and normal caffeine or coffee consumer to provide sample homogeneity. A caffeine consumer was considered as one whose intake was at least 200 mg per day or a person who consumed at least one cup of coffee per day. The caffeine consumption profile was ascertained using the validated self-reported questionnaire [26].

### 2.3. Screening, Inclusion, and Exclusion Criteria

Participants were selected through a controlled screening process based on the set selection criteria. The participants, therefore, went to the Nutrition Clinic of the University of Petra for screening by confirmation of body composition and filling out questionnaires. Furthermore, the participants in this study were only female university students aged 18 to 39 years, in good health, with a minimum BMI of 18.5 and a maximum of 34.9 kg/m^2^. The exclusion criteria included, but were not limited to, acute or chronic medical conditions that could affect the outcome, such as autoimmune diseases, metabolic disorders, cardiovascular or respiratory disorders, and food allergies. Participants currently engaged in another intervention study, those taking in more than 400 mg of caffeine daily, or using weight loss medications and supplements within the last 30 days were excluded. Other exclusions included those with a weight change of more than 10 pounds within the last month, pregnancy, or those who planned pregnancy within the study period, and those who had gastrointestinal surgery with further metabolic consequences. Those who met the above criteria were eligible for participation in the trial with written consent. Each participant was assigned an ID number for confidentiality. In general, the participants were allowed to keep their normal diets and activities as in the usual course of life; however, some preparatory work was carried out before measurements. These included not undertaking strenuous exercise within 24 h of every session, avoidance of caffeine-containing products for at least four days prior to every session as a washout period, and reporting to the testing in a fasting state for at least four hours. Compliance with these guidelines was confirmed before the metabolic assessments.

### 2.4. Body Composition

Measurement of body composition was made at the screening visit for eligibility and documentation of baseline characteristics. Assessments were performed by using a Body Composition Analyzer, BIA 970, Model 1421000139 (InBody Co., Ltd., headquartered in Seoul, Republic of Korea), with calibration to follow the recommendations given by the manufacturer to provide appropriate, accurate, and reliable results. All measurements were taken in the morning after an overnight fast and before any intervention. Standardized conditions have been maintained for all data points. The intervention sessions will be closely scheduled by the research team, taking into consideration the menstrual cycle, where possible, to reduce the variation that may affect body composition measures. The BIA device gave detailed information on the percentage of body fat, lean mass, and total body water. These parameters were recorded and analyzed to track any changes throughout the study period.

### 2.5. Intervention Protocol

**Figure 1:**
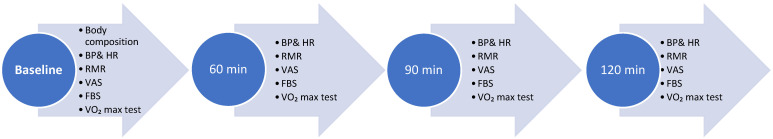
Experimental flow diagram of the study protocol.

Following screening and recruitment, participants were randomized into one of two sequences: a single serving of TC, then a single serving of water (control), or vice versa. Each intervention was conducted under standardized conditions in the Nutrition Clinic, University of Petra, and environmental factors as well as test procedures were thus identical. The participants underwent measurements at four time points: at baseline (t = 0), 60 min, 90 min, and 120 min after beverage consumption as shown in Figure 1. In order to maintain consistency, all trials were conducted at the same time of day for each participant. A trained researcher prepared the TC according to a standardized procedure to ensure that every serving had the same amount of caffeine. Throughout the study, participants were asked to keep their usual dietary and activity habits, though they were instructed to avoid caffeine, alcohol, and high-intensity physical activity for 24 h before each intervention session. The adherence to this was checked by verbal confirmations and by self-reported logs.

### 2.6. Beverage Intervention and Preparation Method 

The TC beans used in this study were obtained from a local supermarket and stored under refrigerated conditions until the day of preparation to maintain freshness. During each experimental session, participants consumed either TC or a control beverage (water). The dose of TC was standardized to 0.136 g/kg body weight, mixed with 55 mL of hot water to provide a dosage of 3 mg/kg body weight. For example, for a participant with a body weight of 50 kg, he/she received 7 g of coffee powder. The TC blend consisted of equal parts of medium and dark roasted Arabica beans, according to the specifications of the manufacturer. Coffee preparation was strictly in accordance with the following methodology: Coffee-to-water ratio was measured with a Toprime Digital Gram Scale (precision ± 0.1 g). Foaming: the mixture has been heated in a traditional cezve for three minutes, reaching 100 °C. Controlling the temperature of the last serving was maintained between the temperatures (95 ± 2 °C) with an HM Digital TDS-3 Handheld Thermometer. Serving glasses were pre-warmed to allow temperature stability within the beverage in the usage period [1,27].

### 2.7. Measurement Procedures

#### 2.7.1. Cardiovascular Measures

##### Resting Heart Rate (HR) and Blood Pressure (BP) Measurements

Resting HR and BP were measured by a fully automated Omron sphygmomanometer (MODEL: M7 Intelli IT(HEM-7361T-EBK), Omron Healthcare Europe, Hoofddorp, The Netherlands, made in Thu Dau Mot City, Vietnam). Measurements were taken under standardized conditions in the clinical setting at the Nutrition Clinic, University of Petra. The participants were seated upright and were asked to remain in this position for at least five minutes before the measurement to ensure that they were fully rested. BP and HR readings were taken from the right arm to ensure consistency across all measurements. Measurements were performed at four different times: at baseline, t = 0, before TC ingestion; 60 min; 90 min; and 120 min after ingestion. Each BP measurement consisted of three consecutive readings, with the average recorded to improve accuracy. All devices used in this study were calibrated every five hours, and all personnel responsible for conducting the measurements underwent standardized training to ensure procedural consistency and minimize variability.

#### 2.7.2. Fasting Blood Sugar (FBS) Measurement

FBS was measured by the Procheck Advance Multi-Functional Monitoring System, Model No. TD4206 (TaiDoc Technology Corporation, New Taipei City, Taiwan). The functionality and batteries of the device were checked before measurement. The finger-prick blood sample for this study was drawn in a standardized way: The fingertip of the participant was sterilized with alcohol, and afterward, a lancet prick on the lateral part of the fingertip drew a small drop of blood. The blood sample was drawn into the test strip, and within seconds, the glucose concentration was displayed on the meter screen. Proper disposal of used test strips and lancets was performed by following local biohazard regulations to ensure safety and compliance with standard protocols.

#### 2.7.3. Gas Exchange Measurements

O_2_ consumption and CO_2_ production were measured by a Desktop Indirect Calorimetry System Cortex MPU31-105 (Cortex Biophysik GmbH, Leipzig, Germany). The subjects were fitted with a facemask connected to a one-way valve, which provided a proper and leak-free seal during measurement. In addition, the metabolic analyzer was calibrated every four hours using reference gases containing 16% O_2_, 5% CO_2_, and nitrogen (N_2_) balance; before each measurement, airflow calibration was performed by using a 3 L syringe, following the manufacturer’s instructions. Before the measurements of baseline resting energy expenditure were obtained, participants were requested to sit quietly for five minutes to allow HR and BP stabilization. They then sat down at an angle for five more minutes before the gas exchange measurement. The metabolic data were measured continuously for 15 min at every assessment time point (baseline, 60 min, 90 min, and 120 min post-ingestion), discarding the first five minutes to allow equilibration. RER was taken as the ratio between CO_2_ produced and O_2_ consumed. Also, values for REE in kcal/day were continuously recorded. The measurement for gas exchange was analyzed in terms of 30 s averages. The participant had to stay awake but seated in a reclined position in a naturally lit room during the measurement, exposed to indirect sunlight, and avoid conversation or extra movement.

#### 2.7.4. Peak Oxygen Consumption (VO_2_ Peak) Testing

Peak oxygen consumption (VO_2_ peak) was determined in an open-circuit spirometry system using the TrueOne 2400^®^ Metabolic Measurement System (Parvo Medics, Inc., Sandy, UT, USA). VO_2_ peak was estimated using an indirect calorimetry system instead of a maximal cardiopulmonary effort test. This approach is widely used in studies with non-athletic participants, as it provides an accurate measure of oxygen consumption while keeping the testing process less demanding and lowering the risks linked to intense exercise. The system was calibrated with room air and known concentrations of gas before each session. VO_2_ peak was derived from breath-by-breath analysis of expired gases. The highest 30 s VO_2_ value was recorded during the test. To confirm attainment of VO_2_ peak, participants had to meet at least two of the following three criteria: reaching 90% of age-predicted maximum HR, reaching a respiratory exchange ratio (RER) > 1.1, and demonstrating a plateau in oxygen uptake is defined as an increase of less than 150 mL·min^−1^ in VO_2_ during the final 60 s. The earlier study tested in our laboratory indicated very satisfying VO_2_ peak test–retest reliability measured by Intraclass Correlation Coefficient (ICC = 0.96) and a Standard Error of Measurement (SEM = 1.4 mL·kg·min^−1^); the mentioned facts just assured the applied measuring procedure about its criterion and reliable state.

### 2.8. Visual Analog Scales

Participants completed 10 cm-anchored VAS at different time points: baseline (t = 0), 60 min, 90 min, and 120 min post-ingestion. Each scale was anchored with “Lowest Possible” on the left and “Highest Possible” on the right. These scales were used to assess subjective responses related to appetite, energy levels, mood, concentration, motivation, sleepiness, and fatigue. This has been previously validated in Arabic and showed a test–retest reliability of r = 0.83, allowing for consistency in subjective assessment [28].

### 2.9. Ethical Approval

The study was conducted between October 2023 and January 2024. The Research Ethics Committee of the Faculty of Pharmacy and Medical Sciences at the University of Petra, Amman, Jordan, granted approval before embarking on the research, with an ethical approval number of E/H/2/8/2023. Before participating in the experiment, participants were explained in detail the experimental procedures involved in the study, risks, and benefits to be derived and were required to give their written informed consent.

### 2.10. Statistical Analysis

Data normal distribution, homogeneity of variance, and sphericity were all checked before statistical analysis. Boxplots were used to identify outliers; extreme values were checked for validity and excluded from analyses if erroneous. In cases of violation of sphericity, analyses were Greenhouse-Geisser corrected. A 2 × 4 repeated-measures ANOVA was used to assess the effects of group (control, case) × time (baseline, 60 min, 90 min, 120 min) on measured variables. In case of a significant interaction, LSD post hoc tests were conducted for pairwise comparisons. Statistical significance was set at *p* ≤ 0.05, and all results are presented as mean ± SD. Statistical analyses were performed using SPSS (version 23, SPSS Inc., Chicago, IL, USA).

## 3. Results

The baseline characteristics of the 52 healthy female university students are summarized in Table 1. Descriptive data indicate a mean age for subjects as 20.25 ± 1.20 years and a mean weight of 63.36 ± 11.79 kg. BMI also showed an average value of 24.44 ± 4.04 kg/m^2^, hence indicating normal BMI.

**Figure 2:**
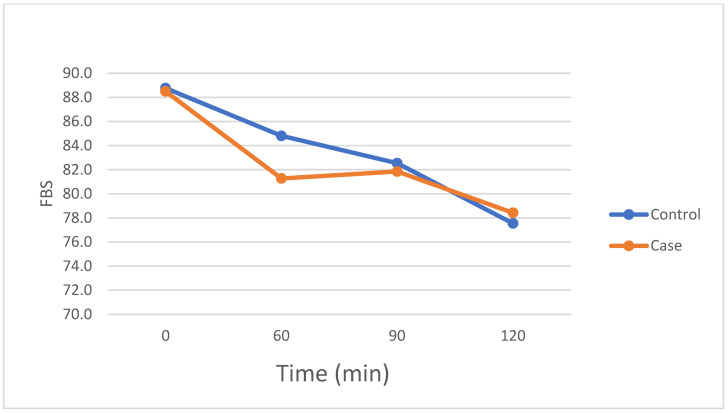
Fasting blood sugar (FBS) response during trials at each time point.

As shown in Table 2, a two-way repeated-measures ANOVA was conducted to examine the effect of different treatments over time on FBS, resting metabolic rate per weight (RMR/Weight), oxygen consumption (VO_2_), and carbon dioxide production (VCO_2_). Analysis of studentized residuals showed the assumption of normality was met, according to the Shapiro–Wilk test, as no significant outliers were identified (i.e., no residuals > ±3 SD). The assumption of sphericity was also met, according to Mauchly’s test of sphericity, for the interaction term, *p* > 0.05. Data are mean ± SD. No significant two-way interaction of group and time was observed for FBS within the statistical significance level: F(3, 75) = 0.995, *p* = 0.40. Again, the main effect of the group showed no statistically significant difference in FBS levels within the control and case groups: F(1, 25) = 0.51, *p* = 0.483. In the case of time, it expressed a significant difference at a *p*-value less than 0.001 (F(2.035, 50.87) = 16.26), so further post hoc analysis at different times was essential. The post hoc analysis revealed significant differences in the control group between baseline and 120 min (*p* < 0.001) and between 60 min and 120 min (*p* < 0.05). In the case group, significant differences were observed between baseline and 60 min (*p* < 0.01), and between baseline and 120 min (*p* < 0.05), as depicted in Figure 2. There were no significant interactions between group and time for RMR/Weight, VO_2_, and VCO_2_: F = 0.157, *p* = 0.93; F = 0.24, *p* = 0.87; and F = 0.009, *p* = 0.999, respectively. No main effect of group comparing groups across time was observed, *p* > 0.05, nor was there a main effect of time comparing time points across groups when examining time, *p* > 0.05, as shown in Table 2.

**Figure 3:**
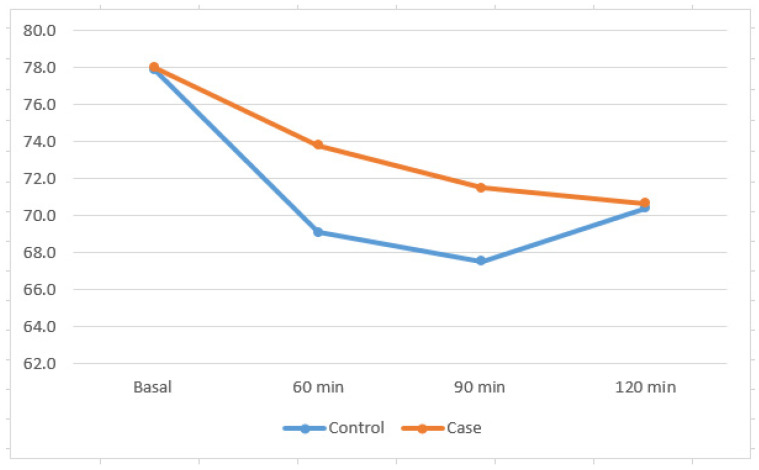
HR response during trials at each time point.

As shown in Table 3, a two-way repeated-measures ANOVA was conducted to compare the effect of treatment and time on SBP and DBP and HR. The Shapiro–Wilk test of studentized residuals suggested that data were normally distributed with no outliers, that is, no residuals greater than ±3 SD. Furthermore, according to Mauchly’s test of sphericity, the assumption of sphericity was met for the interaction term (*p* > 0.05). Data are presented as mean ± standard deviation (SD). There was no significant two-way interaction between the group and time for either SBP or DBP, indicating that longitudinal BP responses for the two groups did not differ. Also, no significant main effect of the group was seen when SBP and DBP were compared over time (*p* > 0.05). Similarly, no main effect of time was seen when time points were compared between groups (*p* > 0.05) (Table 3). For HR, there was no significant two-way interaction between group and time: F(3, 75) = 1.415, *p*-value = 0.245. The main treatment effect did not reveal any difference in HR between groups: F(1, 25) = 5.820, *p*-value = 0.420. The main effect of time showed a statistically significant difference in HR across time points: F(2.035, 50.874) = 28.242, *p* < 0.001 was further analyzed with post hoc pairwise comparisons. Post hoc analysis by LSD correction showed that the control group demonstrated HR at baseline to be significantly higher than that at all the subsequent time points: *p* < 0.05. In the case group, HR was significantly lower in 90 min versus baseline: *p*-value 0.002 and in 120 min versus baseline: *p*-value 0.006 (Figure 3).

### Visual Analog Scale

**Figure 4:**
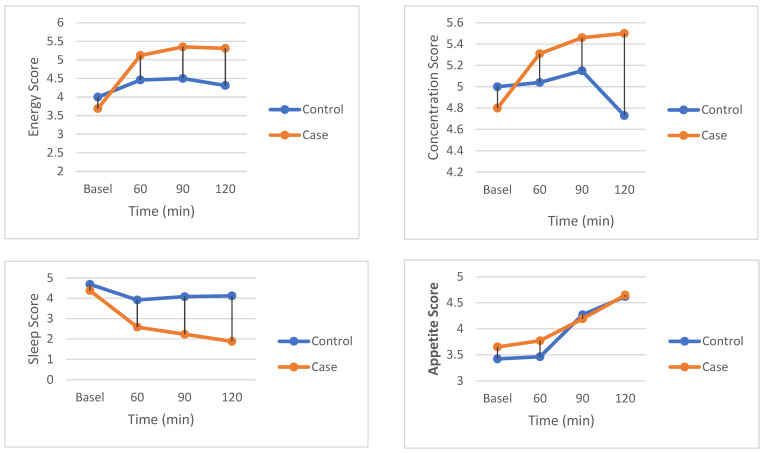
Subjective measures of energy, sleep, and concentration response during trials at each time point. All values are reported as mean ± SD.

As can be seen from Table 4, a two-way repeated-measures ANOVA was conducted to examine the impact of time and treatment on energy, appetite, sleep, mood, concentration, motivation, and fatigue. Studentized residuals analysis confirms normality, as demonstrated with the Shapiro–Wilk test, as there were no outliers, i.e., no residuals greater than ±3 SD. There is homogeneity of variance because Mauchly’s test of sphericity indicates a *p*-value of >0.05. Data are presented as mean ± SD. For energy, there was a statistically significant two-way interaction of group and time: F(3, 75) = 3.02, *p =* 0.035; in the main effect of treatment, no significant difference between the groups was shown: F(1, 25) = 0.536, *p =* 0.471. However, the overall main effect of time revealed a significant difference between time points, F(3, 75) = 7.506, *p* < 0.001, which warranted post hoc pairwise comparisons. No significant differences were observed between time points in the control group (*p* > 0.05); however, the case group had significantly higher energy levels at 60 (*p* < 0.01), 90 (*p* < 0.01), and 120 (*p =* 0.001) minutes compared to baseline. Concerning appetite, a non-significant two-way interaction was obtained between all conditions: F(3, 75) = 0.239, *p =* 0.869 and an insignificant main effect of treatment F(1, 25) = 0.023, *p =* 0.881, while in fact, there was a significant effect with regard to a time factor F(3, 75) = 8.303, *p* < 0.001. Post hoc analysis revealed that appetite at baseline was significantly lower compared to 120 min in both the control group (*p* < 0.001) and the case group (*p* < 0.05), and appetite at 60 min was significantly lower compared to 120 min. With regard to sleep, there was a significant two-way interaction at F = 3.17, *p =* 0.03. There were no significant differences noted in the control group alone. However, in the case group, the sleep score at 60 min was 2.58 ± 2.04 (*p* < 0.05); at 90 min, it was 2.23 ± 1.48 (*p* < 0.05); and at 120 min, it was 1.88 ± 1.45 (*p* < 0.05) and these have significantly fallen with baseline scores of 4.38 ± 2.91. Sleep scores between the control and case groups are significantly different from 90 to 120 min. The mood scores showed a significant interaction of group and time: F = 5.266, *p*-value = 0.030. In the control group, no differences were observed; however, in the case group, mood scores were significantly higher at 60 min (5.15 ± 2.80, *p* < 0.05), 90 min (5.31 ± 3.01, *p* < 0.05), and 120 min (5.42 ± 3.11, *p* < 0.05) when compared with baseline (4.12 ± 2.53). However, no overall significant difference appeared between the control and case groups at any time point. A significant interaction also emerged with regard to concentration, F = 3.263, *p*-value = 0.026. There were no significant changes in the mean across time points in the control group. However, at all-time points in the case group, mean concentration significantly improved from baseline values. In the case of motivation, there was no significant two-way interaction, F(3, 75) = 0.748, *p*-value = 0.527, and nor was the main effect of treatment. However, the main effect of time was significant, F(3, 75) = 6.96, *p* < 0.001. Post hoc analysis indicated that in the case group, motivation at baseline was significantly lower compared to all other time points. Fatigue scores showed a trend of reduction over time following Turkish coffee consumption. While no significant interaction effect was observed, the case group exhibited a slight decline from 3.54 ± 2.95 at baseline to 3.42 ± 2.58 at 60 min, followed by 3.85 ± 2.89 at 90 min and 3.92 ± 2.96 at 120 min. In contrast, the control group demonstrated a more notable decline from 3.27 ± 2.61 at baseline to 2.31 ± 1.81 at 60 min, 2.73 ± 2.20 at 90 min, and 2.62 ± 2.00 at 120 min. These findings indicate a potential mild alertness effect of Turkish coffee, but further research is needed to determine its impact on fatigue perception over time. Figure 4 shows the main outcome variables showing significant changes in subjective measures.

## 4. Discussion

The present randomized crossover-controlled trial was performed to explore the metabolic responses to the consumption of a single dose of TC among healthy university female students. While specific studies focusing solely on Turkish coffee and its effects on female students are limited, the general research on caffeine supports the idea that it can improve alertness, concentration, and possibly academic performance [29,30]. Turkish coffee, being a concentrated source of caffeine, is likely to yield similar benefits. Interested findings emerged in the current study regarding levels of FBS for the intervention participants through different time points of the study. This result may support the debate that coffee may act as a therapeutic agent for type 2 diabetes. While this study focused on the immediate effects of Turkish coffee, the observed drop in FBS supports previous research suggesting that compounds like chlorogenic acid may help regulate glucose by improving insulin sensitivity and reducing liver glucose production [9]. However, it is important to note that blood glucose levels also decreased in the control group, suggesting that factors beyond coffee consumption, such as fasting duration, individual metabolic differences, or the body’s natural regulatory mechanisms, may have contributed to this outcome. Future research should explore these aspects to provide a more comprehensive understanding of coffee’s role in glucose metabolism. More research is also needed through extended intervention studies to determine the long-term impact of these effects. A trend of descending values of FBS has been shown starting from the baseline until the last time point of the study. The caffeine in this single dose of TC is thought to have a blood sugar-dropping effect. This effect was consistent with a study conducted by Yusni and Yusuf [31]. The authors documented that caffeine of the Gayo Arabica black coffee enhances blood sugar reduction and, furthermore, provided a possible explanation of the effect through a substantial influence of caffeine on the glycemic response by causing a slight increase in the levels of insulin and a decrease in cortisol levels. Another explanation that supports our results has been shown through the findings of a study by Ballis [32]. The author of the foregoing study researched that coffee is rich in the content of soluble fibers, the main ones being type II arabinogalactans and galactomannans, reducing glucose absorption into the blood and improving control over blood sugar levels. Another reason that was accounted in a study [18] was that evidence indicates the active presence of the element of Trigonelline and coffee polyphenol, preventing the release of glucose in the liver, enhances peripheral glucose uptake. They also documented that coffee contains chlorogenic acid (CGA) that activates adenosine monophosphate-activated protein kinase (AMPK), which inhibits fatty acid synthesis and enhances the production of hepatic glucose. Despite all the previous explanations that assure our study results, other studies revealed results contrasted with our results. Church and colleagues [6] reported that both participants ingested caffeinated TC and decaffeinated coffee (DC) 60 min prior to the 5 km time trial and had significantly elevated plasma glucose during all times. Moreover, two previous studies conducted [33,34] revealed that caffeine elevates epinephrine levels, which induces insulin resistance and causes a significant increase in blood glucose concentrations. Another two studies [35,36] also confirmed the negative impact of caffeine on blood glucose concentration; researchers indicated that caffeine decreases insulin sensitivity index and, consequently, increases glucose concentration compared to placebo. The variability in blood glucose response to coffee consumption across studies may be attributed to multiple factors, including differences in individual caffeine metabolism, genetic predisposition, habitual coffee consumption, and insulin sensitivity. Caffeine has been shown to acutely increase catecholamine release, potentially leading to transient insulin resistance, whereas chlorogenic acid, a key polyphenol in coffee, is thought to enhance glucose uptake and reduce hepatic glucose production. However, the interplay between these mechanisms varies based on factors such as dosage, timing, and individual metabolic conditions. These discrepancies highlight the complexity of coffee’s impact on glucose metabolism and emphasize the need for further controlled studies to clarify its long-term metabolic effects [18,37,38].

Another result in our study was regarding the effect of TC in reducing HR from a basal time point of the study until the ending point with no significant change in SBP or DBP. The relationship between coffee intake and cardiovascular health is still controversial, and no previous study agrees with our study. From a pathophysiological point of view, Ref. [39] reported that the hemodynamic effects of caffeine are associated with increased vascular resistance and vasoconstriction. They investigated the mechanisms beyond this effect and reported that these mechanisms include (1) inhibiting phosphodiesterases and thus increasing cyclic AMP and cyclic GMP levels, (2) antagonistic effects on adenosine receptors by blocking A1 and A2 receptors, (3) increasing the plasma concentrations of catecholamines due to activation of the sympathetic nervous system, (4) increasing of stress-like in plasma adrenocorticotropin (ACTH) and cortisol concentrations through stimulation of adrenal cortex, (5) activation of the renin–angiotensin–aldosterone system through renal effects of caffeine, and (6) mobilization of intracellular calcium which increases caffeine-induced vasoconstriction. Ref. [40] found that there was an insignificant difference in BP and HR between participants taking coffee containing a high rate of caffeine (TC) and the other DC. Similarly, Ref. [41] found that there were insignificant differences in the influence of caffeinated or DC on BP or HR. Furthermore, Ref. [42] indicated that an insignificant change in HR neither in habitual nor non-habitual coffee consumers. They also documented that intake of coffee may disturb the measurement of BP if the measurement is made within a few hours after drinking, and this could explain why measurements of BP were not changed in our study. In the same context, Ref. [5], in their investigation among young adults, indicated that acute coffee intake after moderate-intensity aerobic exercise enhances the delaying of parasympathetic recovery with no influence on the respiratory rate. On the other hand, Kristian and colleagues [43] documented the opposite result of our study. Researchers reported that caffeine among elite male endurance athletes causes increased maximal HR (HR_peak_). This is attributed to the increase in the body’s need for increasing the maximal oxygen uptake in elite athletes. In similar studies, researchers [43,44] documented elevated HRs among athletes after caffeine treatment.

The role of TC consumption in enhancing or reducing appetite among participants was one of the crucial subjective measures between the two groups of the study. The enhancing ability of coffee appetite among participants was shown between different time points of the study. This effect of coffee is noteworthy, especially for a person who suffers from anorexia and poor appetite feelings. Generally, the results of different studies regarding the impact of coffee consumption, food intake, and appetite are areas of contradicting findings. In a study conducted by Gavrieli and colleagues [45], they found that a moderate intake of coffee enhanced the reduction in energy intake compared to lower or no coffee intake. Furthermore, Gavrieli and colleagues [46] indicated that caffeinated coffee does not affect energy intake and appetite. Schubert et al. [47] demonstrated that coffee intake 3–4.5 h before a meal was associated with minimal influence on food and macronutrient intake and that coffee intake 0.5–4 h before a meal was associated with suppression of acute energy intake [47]. The reasons beyond differences in the association between coffee and caffeine intake and appetite may be partially explained by their influences on physiological and psychological mediators of food consumption [48]. These influences may be responsible for alterations in gut hormones, gastric emptying, and energy intake [49]. From a physiological point of view, Ref. [50] investigated the impact of caffeinated coffee on both hormones leptin and ghrelin. They found that no significant differences in the leptin levels were found between the treatments of caffeinated coffee and decaffeinated coffee. Meanwhile, they documented that the caffeinated coffee treatment led to lower plasma ghrelin as compared to the DC treatment. This effect emphasizes that caffeinated coffee manipulated the level of ghrelin towards an anorexigenic effect.

Participants in our study consuming TC experienced significantly greater concentration, motivation, mood, and feelings of energy. The latter may consequently result in a significant improvement in performance. Researchers [51] reported that caffeine acts on nerve cells and increases their activities so that it can enhance the reduction of fatigue, increase feelings of energy, increase alertness, and increase concentration. Our results were not in accordance with the results of the study conducted by [43], which documented that there were no differences regarding self-reported “current fitness” and motivation between caffeine and placebo trials among elite male endurance athletes. On the contrary, the results of our study were in agreement with the results of a study conducted by [6], which revealed that TC ingestion is associated with a significant increase in subjective feelings of energy, alertness, and focus. Furthermore, ref. [52] reported that caffeinated coffee was associated with higher alertness scores. In addition, a Turkish study [53] investigated that receiving moderate amounts of caffeine-containing drinks was associated with positive benefits, such as increased motivation, alertness, and feeling energetic. Moreover, ref. [54] reported that acute caffeine doses are highly beneficial for numerous psychomotor variables, including mood state. In the same context, Makki and colleagues [55] have recently documented that there is an insignificant association between the severity of depression, anxiety, and stress and caffeine consumption among university students and that high intake of caffeine is not correlated with high levels of depression, stress, and anxiety. From a performance point of view, five studies in the last ten years documented that intake of TC is highly associated with higher sports performance [56,57,58]. Moreover, a recent crossover trial among elite e-sports players found that 3 mg/kg of caffeine supplementation improved cognitive abilities and shooting performance among study participants [59]. Physiologically, the body does not get tired after caffeine consumption before exercise due to caffeine’s ergogenic effect, which increases physical performance by three mechanisms: (1) increasing the intracellular calcium mobility, (2) increasing the free fatty acid burning, and (3) working as an adenosine receptor antagonist [60].

Based on the effect of TC on the factors in the later paragraph, it was highly important to find out the association between acute doses of TC and sleep among participants. Results of our study emphasize the role of this coffee in reducing sleep scores among participants in the intervention group through different time points of the study and even between participants of both the intervention group and control group. In agreement with our results, Alhabee and colleagues [41] demonstrated an association between insomnia and high doses of TC. This relationship is based on the outcomes that include increased sleep disruption and sudden or frequent awakening. A recent study [61] revealed that caffeine in coffee is a commonly psychoactive substance that affects sleep quality. They found that there was a significant positive correlation between the intake of caffeine and poor sleep quality. Moreover, the results of our study corroborate with other studies, which indicated that coffee interferes with sleep by negatively affecting sleep duration, total sleep time, sleep latency, and sleep quality [62,63,64,65]. However, this study has certain limitations that should be considered. The non-blinded design may have introduced expectation bias, particularly in subjective outcomes such as appetite and sleep perception. While this enhances ecological validity, future research should implement a placebo-controlled, double-blind approach to further isolate physiological effects from potential psychological influences. Additionally, inter-individual variability in caffeine metabolism was not assessed. Factors such as genetic predisposition, habitual coffee intake, and cultural variations may have influenced participants’ responses to TC. Baseline caffeine tolerance could have also played a role in modifying cardiovascular and metabolic outcomes. In addition, one of the limitations of our study is that we did not control for menstrual cycle phases, which may have influenced metabolic and cardiovascular responses due to hormonal fluctuations. Given the relatively small sample size, this variability might have affected the observed outcomes. Future studies should consider menstrual cycle tracking to refine the understanding of how TC influences physiological parameters across different hormonal phases. Moreover, future studies should consider stratifying participants based on caffeine metabolism profiles to further explore these individual differences. Furthermore, the study was conducted in a homogeneous sample of young, healthy female participants, which may limit the generalizability of the findings to males, older adults, or individuals with metabolic conditions. Expanding the sample population would provide a broader understanding of TC’s effects across different demographics. Finally, this study focused on the acute effects of a single serving of TC, which does not capture the long-term implications of habitual consumption. Future research should investigate repeated intake over extended periods to assess its sustained impact on cardiovascular and metabolic health. Addressing these limitations in future studies will enhance the applicability of the findings and provide deeper insights into the potential health benefits and risks of TC consumption.

## 5. Conclusions

This study sets out to explore how a single serving of Turkish coffee (TC) affects heart rate, metabolism, and other physiological responses in healthy female university students. The results show that TC had a noticeable impact on HR, appetite, and sleep patterns. Specifically, drinking TC led to a slower HR, reduced appetite, and lower sleep scores compared to those who drank only water. However, BP and FBS levels did not show significant changes. These findings suggest that TC has effects beyond just its caffeine content, influencing how the body responds in the short term. Given how popular TC is, more research is needed to understand its long-term effects on metabolism and overall health.

## Figures and Tables

**Table 1:** Baseline subject characteristics (n = 52).

Characteristic	Mean Value
Age (years)	20.25 ± 1.20
Weight (kg)	63.36 ± 11.79
BMI (kg/m^2^)	24.44 ± 4.04

All values are mean ± SD. BMI, Body Mass Index.

**Table 2:** Effect of oral administration of a single cup (250 mL) of coffee beverage on FBS, RMR/Weight, VO_2_, and VCO_2_ among healthy university female students (n = 52).

Group	FBS (mg/dL)			
	Baseline	60 min	90 min	120 min
Control	88.77 ± 7.80 ^aA^	84.81 ± 8.08 ^abA^	82.54 ± 9.72 ^bcA^	77.54 ± 12.44 ^cA^
Case	88.50 ± 6.79 ^aA^	81.27 ± 8.67 ^bA^	81.85 ± 10.24 ^bA^	78.42 ± 11.38 ^bA^
	**RMR/Weight (kcal/d/kg)**			
Control	28.11 ± 3.82 ^aA^	27.12 ± 4.86 ^aA^	27.12 ± 4.82 ^aA^	27.04 ± 4.47 ^aA^
Case	28.45 ± 4.91 ^aA^	29.50 ± 5.89 ^aA^	29.02 ± 5.16 ^aA^	28.76 ± 5.08 ^aA^
	**VO_2_ (L/min)**			
Control	0.25 ± 0.04 ^aA^	0.24 ± 0.05 ^Aa^	0.24 ± 0.05 ^aA^	0.24 ± 0.05 ^aA^
Case	0.25 ± 0.07 ^aA^	0.26 ± 0.05 ^Aa^	0.26 ± 0.06 ^aA^	0.26 ± 0.06 ^aA^
	**VCO_2_ (L/min)**			
Control	0.23 ± 0.05 ^aA^	0.22 ± 0.07 ^Aa^	0.21 ± 0.06 ^aA^	0.21 ± 0.05 ^aA^
Case	0.26 ± 0.09 ^aA^	0.24 ± 0.07 ^Aa^	0.24 ± 0.07 ^aA^	0.24 ± 0.07 ^aA^

Values represent means ± SD. Means with the same lowercase letter, in a row, and same uppercase letter, in a column, are not significantly different (*p* > 0.05). RMR, resting metabolic rate; VCO_2_, carbon dioxide production; VO_2_ max, maximum oxygen consumption; FBS, fasting blood sugar.

**Table 3:** Effect of oral administration of a single cup (250 mL) of coffee beverage on systolic and diastolic (BP) and HR among healthy university female students (n = 52).

Group	Systolic and Diastolic (BP) (mmHg)			
	Basal	60 min	90 min	120 min
	**SBP**			
Control	104.69 ± 12.33 ^aA^	98.08 ± 11.63 ^aA^	97.12 ± 13.39 ^aA^	97.92 ± 11.47 ^aA^
Case	103.27 ± 12.50 ^aA^	100.04 ± 14.04 ^aA^	98.46 ± 12.42 ^aA^	101.96 ±3.29 ^aA^
	**DBP**			
Control	73.23 ± 8.90 ^aA^	69.62± 6.66 ^aA^	68.23 ± 8.80 ^aA^	67.96 ± 8.19 ^aA^
Case	77.88 ± 9.98 ^aA^	72.46 ± 9.94 ^aA^	71.62 ± 9.33 ^aA^	72.54 ± 13.49 ^aA^
	**HR (bpm)**			
Control	77.88 ± 9.98 ^aA^	69.12 ± 7.93 ^bA^	67.54 ± 9.49 ^bA^	70.38 ± 10.60 ^bA^
Case	78.0 ± 10.21 ^Aa^	73.81 ± 9.87 ^abA^	71.50 ± 9.48 ^bA^	70.65 ± 10.11 ^bA^

Values are expressed as mean ± SD. Means for treatments not sharing a common lowercase letter (within a row) or uppercase letter (within a column) are significantly different, *p* ≤ 0.05. SBP, systolic blood pressure; DBP, diastolic blood pressure; HR, heart rate.

**Table 4:** Effect of oral administration of a single cup (250 mL) of coffee beverage on VAS among healthy university female students (n = 52).

Group	Energy			
	Basal	60 min	90 min	120 min
Control	4.00 ± 2.67 ^aA^	4.46 ± 2.35 ^aA^	4.50 ± 2.61 ^aA^	4.31 ± 3.00 ^aA^
Case	3.69 ± 2.56 ^aA^	5.12 ± 3.05 ^bA^	5.35 ± 3.03 ^bA^	5.31 ± 3.09 ^bA^
	**Appetite**			
Control	3.42 ± 2.86 ^aA^	3.46 ± 2.67 ^aA^	4.27 ± 2.99 ^abA^	4.62 ± 2.98 ^bA^
Case	3.65 ± 2.70 ^aA^	3.77 ± 2.75 ^aA^	4.19 ± 2.98 ^abA^	4.65 ± 3.21 ^abA^
	**Sleep**			
Control	4.69 ± 3.27 ^aA^	3.92 ± 2.81 ^aA^	4.08 ± 2.93 ^aA^	4.12 ± 3.02 ^aA^
Case	4.38 ± 2.91 ^aA^	2.58 ± 2.04 ^bA^	2.23 ± 1.48 ^bB^	1.88 ± 1.45 ^bB^
	**Mood**			
Control	4.88 ± 3.04 ^aA^	4.96 ± 3.0 ^aA^	4.77 ± 2.85 ^aA^	4.96 ± 2.72 ^aA^
Case	4.12 ± 2.53 ^aA^	5.15 ± 2.80 ^bA^	5.31 ± 3.01 ^bA^	5.42 ± 3.11 ^bA^
	**Concentration**			
Control	5.00 ± 2.77 ^aA^	5.04 ± 2.76 ^aA^	5.15 ± 2.88 ^aA^	4.73 ± 2.97 ^aA^
Case	4.80 ± 2.74 ^aA^	5.31 ± 2.90 ^bA^	5.46 ± 2.89 ^bA^	5.50 ± 3.10 ^bA^
	**Motivation**			
Control	4.27 ± 2.13 ^aA^	4.96 ± 2.72 ^aA^	4.77 ± 2.62 ^aA^	4.81 ± 2.65 ^aA^
Case	4.04± 2.6 ^aA^	4.88 ± 2.83 ^bA^	5.08 ± 2.67 ^bA^	5.19 ± 3.02 ^bA^
	**Fatigue**			
Control	3.27 ± 2.61 ^aA^	2.31 ± 1.81 ^aA^	2.73 ± 2.20 ^aA^	2.62 ± 2.00 ^aA^
Case	3.54 ± 2.95 ^aA^	3.42 ± 2.58 ^aA^	3.85 ± 2.89 ^aA^	3.92 ± 2.96 ^aA^

Values are the mean ± SD. Numbers followed by the same letter (small script) in the same row and numbers followed by the same letter (large script) in the same column are not significantly different (*p* > 0.05).

## Data Availability

The data presented in this study are available on request from the corresponding author due to (privacy, legal or ethical reasons).

## Abbreviations

ACTH	Adrenocorticotropic Hormone
AMP	Adenosine Monophosphate
AMPK	Adenosine Monophosphate-Activated Protein Kinase
ANOVA	Analysis of Variance
BIA	Bioelectrical Impedance Analysis
BMI	Body Mass Index
BP	Blood Pressure
CGA	Chlorogenic Acid
DC	Decaffeinated Coffee
DBP	Diastolic Blood Pressure
FBS	Fasting Blood Sugar
GMP	Guanosine Monophosphate
HPA axis	Hypothalamic–Pituitary–Adrenal Axis
HR	Heart Rate
ICC	Intraclass Correlation Coefficient
RER	Respiratory Exchange Ratio
REE	Resting Energy Expenditure
RMR	Resting Metabolic Rate
SBP	Systolic Blood Pressure
SEM	Standard Error of Measurement
SPSS	Statistical Package for the Social Sciences
TC	Turkish Coffee
VAS	Visual Analog Scale
VCO_2_	Carbon Dioxide Production
VO_2_ max	Maximum Oxygen Consumption
